# Editorial: Organ crosstalk and other responses to an activated immune system in trauma and disease

**DOI:** 10.3389/fimmu.2025.1651022

**Published:** 2025-07-23

**Authors:** Martijn van Griensven, Elizabeth R. Balmayor, Frank Hildebrand

**Affiliations:** ^1^ Department Cell Biology-Inspired Tissue Engineering (cBITE), MERLN Institute for Technology-Inspired Regenerative Medicine, Maastricht University, Maastricht, Netherlands; ^2^ Experimental Orthopaedics and Trauma Surgery, RWTH Aachen University Hospital, Aachen, Germany; ^3^ Department of Orthopaedics, Trauma and Reconstructive Surgery, RWTH Aachen University Hospital, Aachen, Germany

**Keywords:** organ crosstalk, immune system, trauma, tissue regeneration, *in vitro* models, *in vivo* models

Understanding how the immune system interacts with other organs is essential for addressing trauma, degenerative diseases, and tissue regeneration. This requires focused attention not only on the immune system’s elements (e.g., proteins, receptors, and cell types) but also on the tissues and organs involved in the clinical condition under consideration. The immune system does not act in isolation — it communicates with virtually all tissues via cytokines, extracellular vesicles, pattern recognition receptors, and a wide range of cellular actors. At the tissue cellular level, immune cells communicate by humoral factors to cells of the bone, the endocrine, gastrointestinal, and nervous systems. These signals can be responsible for both, protective and pathogenic outcomes across multiple organ systems.

With this Research Topic, entitled *Organ Crosstalk and Other Responses to an Activated Immune System in Trauma and Disease*, we aimed to capture emerging mechanistic insights and translational advances in this multifaceted field. We are proud to present a Research Topic of 11 high-quality contributions, among which are 10 original research articles and 1 narrative review. These articles cover *in vitro* models, animal studies, and clinical data from trauma and immunological research.

Several studies focused on joint and skeletal tissues, where the intersection of inflammation and regeneration is critical. Smith et al. developed a novel *in vitro* micro-physiological system that models cartilage-bone crosstalk under inflammatory conditions, offering a platform for studying arthritis mechanisms. Ruths et al. expanded on this by showing that terminal complement complex - also known as TCC – has a crucial role by accelerating chondrocyte senescence, linking immune dysregulation to osteoarthritis progression in aging and trauma. Gaining insight into the mechanisms underlying chondrocyte senescence and their associated secretory phenotype could be pivotal for advancing novel therapeutic approaches to osteoarthritis.

The impact of chronic alcohol consumption on immune biomarkers in trauma patients was investigated by Hammour et al., who found altered cytokine patterns that undermine the reliability of traditional markers like C-reactive Protein, highlighting the need for personalized diagnostic tools. The data presented in this study indicate that patient-specific approaches may be necessary in cases of alcoholized trauma patients, not only for treatments but also for the possible prediction of associated complications.

Focusing on neurological injury, Horauf et al. demonstrated that extracellular vesicles carrying specific surface markers, such as CD47, CD56, CD68, and ADAM17, correlate with neurological outcomes in traumatic spinal cord injury, revealing potential diagnostic avenues.

The liver’s immunological role was highlighted by Li et al., who elegantly showed that combined inhibition of C5 and CD14 modulates the RANK-RANKL-OPG axis after polytrauma, revealing a liver-bone-immune interface and offering potential targets to reduce systemic inflammation and hepatic injury. Another contribution by Zhou et al. also investigated the combined inhibition of C5 and CD14. The work they presented investigated the effect of the surgical invasiveness on pulmonary microRNA expression in a porcine polytrauma model. Particular emphasis was placed by the authors on including miRNAs with a known role in respiratory failure and/or lung pathologies. Interestingly, the authors showed that the combination of an early total care surgical procedure with a combined C5 and CD14 inhibition therapy resulted in beneficial terms of pulmonary microRNA expression. Their findings suggest that combining surgical trauma with an immune modulation drug promotes anti-inflammatory, regenerative miRNA profiles.

Acute kidney injury, also known as AKI, is among the systemic effects associated with multi-organ dysfunction in polytrauma. In fact, AKI is one of the most common and serious complications in trauma, contributing to high morbidity and mortality. Yang et al. provided compelling evidence that early elevation of high-mobility group box 1 (HMGB1) levels predicts AKI and multiple organ failure up to 12 hours before classical markers like creatinine, making it a promising early biomarker for critical trauma care.

In the field of bone regeneration, Penna-Martinez et al. showed that CD8^+^ lymphocyte depletion from bone marrow mononuclear cells significantly improved healing in a rat femoral defect model, underscoring the impact of immune cell composition on regenerative strategies. Naturally, it can be expected that depletion of CD8^+^ lymphocytes impacts both the inflammatory response as well as the bone remodeling process during healing. The relevant findings presented by this work could be immediately translatable to patient care as part of the advances in cellular therapeutics for bone healing.

Kristiansen et al. revealed that femoral nailing can trigger bone marrow emboli that, in turn, may provoke a systemic IL-6 response and localized inflammation in the lung and heart, calling attention to immunological risks during orthopedic procedures.

From a clinical perspective, Becker et al. demonstrated that elevated extracellular particles in plasma could be used as a predictor of in-hospital mortality after severe trauma, providing a powerful biomarker for patient stratification at emergency admission. The implementation of such a biomarker will undoubtedly catapult the detection of possible polytraumatized patient complications at early stages to a completely new level.

The Research Topic also includes a narrative review, elegantly written by Ganse, who systematically assesses clinical methods to accelerate fracture healing, including physical stimulation, growth factors, and hormonal therapies. This review also identifies knowledge gaps in clinical translation and underscores the importance of standardized outcome measures.

Taken together, these contributions provide a rich and multidisciplinary overview of the immune system’s interactions with organ systems during trauma, degeneration, and repair. They highlight the clinical potential of immunological biomarkers, experimental modeling of inter-organ communication, and targeted immunomodulation in regenerative strategies. As Topic Editors, we hope this Research Topic will catalyze future interdisciplinary studies and translational innovation in trauma immunology. We are thrilled to note that, as of the date this editorial was written, the Research Topic has received over 26,000 views and a total of 6,632 downloads. These figures reflect the significant impact of our Topic on the readership of Frontiers in Immunology and the wider scientific community.

Ultimately, only through the combined research efforts of scientists and clinicians, we will be able to deliver improved care and solutions for critically ill patients. We hope the work presented in this Research Topic contributes meaningfully to advancing the field.

We thank all contributing authors and reviewers for their rigorous work and insightful contributions.

